# Oxidation of peroxiredoxin-4 induces oligomerization and promotes interaction with proteins governing protein folding and endoplasmic reticulum stress

**DOI:** 10.1016/j.jbc.2021.100665

**Published:** 2021-04-23

**Authors:** Evan A. Elko, Allison M. Manuel, Sheryl White, Ester Zito, Albert van der Vliet, Vikas Anathy, Yvonne M.W. Janssen-Heininger

**Affiliations:** 1 Department of Pathology and Laboratory Medicine, University of Vermont, Burlington, Vermont, USA; 2 Department of Neurological Sciences, University of Vermont, Burlington, Vermont, USA; 3 Department of Biochemistry and Molecular Pharmacology, Istituto di Ricerche Farmacologiche Mario Negri IRCCS, Milan, Italy

**Keywords:** airway epithelial cells, ER stress, lung, peroxiredoxin-4, ER, endoplasmic reticulum, ERO1, ER oxidoreductin 1, ERO1a, endoplasmic reticulum oxidoreductase alpha, ERP44, endoplasmic reticulum resident protein 44, H_2_O_2_, hydrogen peroxide, HMW, high molecular weight, HSPA5, heat shock 70 kDa protein 5, IPF, idiopathic pulmonary fibrosis, MW, molecular weight, NEM, *N*-ethyl maleimide, P4HB, prolyl 4-hydroxylase subunit beta, PDI, protein disulfide isomerase, PDIA6, protein disulfide isomerase A6, PRDX, peroxiredoxin, SBP, streptavidin-binding peptide, TBuOOH, tertbutyl hydroperoxide, TXNDC5, thioredoxin domain–containing protein 5, UPR, unfolded protein response

## Abstract

Peroxiredoxins (PRDXs) catalyze the reduction of hydrogen peroxide (H_2_O_2_). PRDX4 is the only peroxiredoxin located within the endoplasmic reticulum (ER) and is the most highly expressed H_2_O_2_ scavenger in the ER. PRDX4 has emerged as an important player in numerous diseases, such as fibrosis and metabolic syndromes, and its overoxidation is a potential indicator of ER redox stress. It is unclear how overoxidation of PRDX4 governs its oligomerization state and interacting partners. Herein, we addressed these questions *via* nonreducing Western blots, mass spectrometry, and site-directed mutagenesis. We report that the oxidation of PRDX4 in lung epithelial cells treated with tertbutyl hydroperoxide caused a shift of PRDX4 from monomer/dimer to high molecular weight (HMW) species, which contain PRDX4 modified with sulfonic acid residues (PRDX4-SO_3_), as well as of a complement of ER-associated proteins, including protein disulfide isomerases important in protein folding, thioredoxin domain–containing protein 5, and heat shock protein A5, a key regulator of the ER stress response. Mutation of any of the four cysteines in PRDX4 altered the HMW species in response to tertbutyl hydroperoxide as well as the secretion of PRDX4. We also demonstrate that the expression of ER oxidoreductase 1 alpha, which generates H_2_O_2_ in the ER, increased PRDX4 HMW formation and secretion. These results suggest a link between SO_3_ modification in the formation of HMW PRDX4 complexes in cells, whereas the association of key regulators of ER homeostasis with HMW oxidized PRDX4 point to a putative role of PRDX4 in regulating ER stress responses.

Peroxiredoxins (PRDXs) are a family of highly expressed proteins that scavenge cellular hydrogen peroxide (H_2_O_2_). PRDXs also play critical roles in redox relays in which they oxidize specific client proteins, thereby relaying signals from H_2_O_2_ or related species to select protein targets. Because of their rapid reaction with H_2_O_2_ and abundant expression in different cellular compartments, PRDXs play a critical role in maintaining localized redox homeostasis (1) and redox signaling *via* the oxidation of downstream target proteins (2). PRDXs regulate multiple physiological functions, including circadian rhythm, cell death, and cell cycle progression (3, 4, 5, 6). The PRDX family can be divided into groups based on the mechanism by which they metabolize H_2_O_2_. PRDX1, PRDX2, PRDX3, and PRDX4 are 2-cys PRDXs, whereas PRDX5 is an atypical 2-cys PRDX and PRDX6 is a 1-cys PRDX. The 2-cys PRDXs contain a highly reactive peroxidatic cysteine, which interacts with H_2_O_2_ to release water and subsequently forms a disulfide bond between the peroxidatic cysteine and the spatially adjacent resolving cysteine. PRDXs can also be inactivated by overoxidation. If H_2_O_2_ levels increase excessively, a second H_2_O_2_ molecule can interact with an oxidized peroxidatic cysteine before the intermediate disulfide bond forms, thus overoxidizing the PRDX to the sulfinic (SO_2_) and sulfonic (SO_3_) forms. Overoxidation of PRDXs has been shown to increase the stability of high molecular weight (HMW) decamers of PRDXs and thus causes a shift in the cellular PRDX pool from monomeric and dimeric species to HMW decamers or dodecamers (7). PRDX oligomers, specifically overoxidized PRDXs, are believed to act as chaperones similar to heat shock proteins (8, 9); however, the exact nature of the chaperone activity and the proteins that PRDX decamers or dodecamers interact with in this chaperone capacity have yet to be fully studied.

The fourth member of the family, PRDX4, is the only PRDX found in the endoplasmic reticulum (ER). The redox poise of the ER is vital to many cellular processes, including the formation of disulfide bounds in naive proteins. The ER is more oxidizing than the cytosol, and its redox balance is tightly controlled by glutathione and oxidoreductase enzymes, such as glutathione peroxidases 7 and 8 and PRDX4 (10). In humans, PRDX4 is the most highly expressed H_2_O_2_-scavenging protein in the ER. It has been shown to be a potential component of a novel disulfide bond–generating process that relies on a redox relay between H_2_O_2_, PRDX4, and protein disulfide isomerases (PDIs) to keep PDIs oxidized, as opposed to the classical model of ER oxidoreductin 1 (ERO1) recycling of PDIs (11, 12). The exact role of PRDX4 in ER redox homeostasis and redox signaling remains to be fully elucidated.

When first discovered, it was believed that PRDX4 was a secreted protein because of its N-terminal signal peptide targeting it for translation into the ER and lack of an ER retention (KDEL) sequence (13). However, later studies show that PRDX4 is maintained in the ER through interactions with various ER proteins such as endoplasmic reticulum resident protein 44 (ERP44) (14), and the latter interaction is believed to be redox dependent as a disulfide bond forms between PRDX4 and ERP44. PRDX4 has been detected in the extracellular environment where it has been proposed as a potential biomarker of various diseases (15, 16, 17).

The role of PRDX4 overoxidation in governing ER redox homeostasis remains incompletely understood. Notably, the role of oxidation of specific cysteines in regulating oligomerization, interaction of PRDX4 with its client proteins, and its ability to be retained in the ER also are not completely known. Therefore, in this article, we sought to address the effect of PRDX4 overoxidation on oligomerization, its interactome, and secretion. Our findings indicate that PRDX4 HMW species interact with ER-associated proteins (thioredoxin domain–containing protein 5 [TXNDC5], ERP44, protein disulfide isomerase A6 [PDIA6], protein disulfide isomerase (prolyl 4-hydroxylase subunit beta [P4HB]), and ER chaperone binding immunoglobulin protein (heat shock 70 kDa protein 5 [HSPA5]) as well as the cytosolic localized PRDX1). Cysteines in PRDX4 play an important role in the formation of HMW species, and oxidation-resistant mutants modeled on other PRDX4 family members alter PRDX4 HMW species and secretion from lung epithelial cells.

## Results

### Overoxidation of PRDX4 leads to increased HMW species

**Figure 1:**
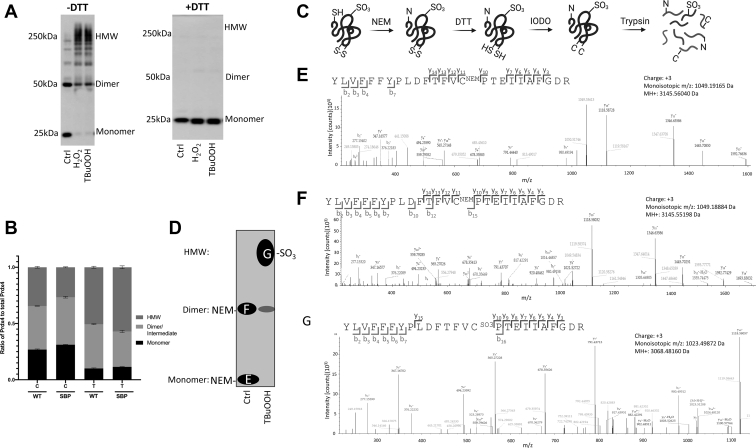
**Overoxidation of PRDX4 leads to increased HMW species.***A*, Western blot for PRDX4 in C10 cells expressing WT PRDX4 treated with 500 μM hydrogen peroxide (H_2_O_2_) or 500 μM tertbutyl-hydroperoxide (Tert) for 2 min. *B*, quantification of PRDX4 monomer, dimer, and HMW species in WT PRDX4 (WT) and SBP-tagged PRDX4 (SBP) treated with vehicle control (C) or 500 μM TBuOOH (T). *C*, diagram of protein cysteine labeling scheme for MS (created with BioRender.com). *D*, schematic showing PRDX4 Western blot bands that were excised for the corresponding MS/MS spectra. *E*–*G*, MS/MS spectra for the peptide containing the peroxidatic cysteine in PRDX4 for the oxidized HMW species and steady-state monomer and dimer species, respectively. HMW, high molecular weight; MS/MS, tandem MS; PRDX4, peroxiredoxin-4; SBP, streptavidin-binding peptide.

H_2_O_2_ and tertbutyl hydroperoxide (TBuOOH) have been shown to be substrates for mammalian PRDXs and are capable of inducing inactivation *via* overoxidation (18). In order to determine the effect of the overoxidation of PRDX4 on its oligomerization, we treated mouse lung epithelial cells (C10) with H_2_O_2_ or TBuOOH. Treatment with H_2_O_2_ or TBuOOH led to substantial increases in HMW species of PRDX4 as well as a loss of PRDX4 monomer and decreased PRDX4 dimer species using nonreducing PAGE (DTT; Fig. 1*A*, *left panel*). Oxidation by either H_2_O_2_ or TBuOOH showed identical shifts toward HMW species. As a previous study showed TBuOOH to be a more potent inactivator of rat PRDX4 (19), we chose to use TBuOOH as the substrate by which to induce PRDX4 overoxidation for the remainder of the studies. All PRDX4 HMW species and dimers were susceptible to reduction by DTT, and the same samples run nonreducing showed only the monomer form of PRDX4 (Fig. 1*A*, *right panel*). Expression of streptavidin-binding peptide (SBP)–tagged PRDX4 (PRDX4-SBP) in C10 cells allows for the pulldown and detection of PRDX4 oxidation status of the peroxidatic cysteine in its monomer, dimer, and HMW forms by tandem MS (MS/MS). The addition of the SBP tag to PRDX4 did not alter the oligomerization compared with WT-PRDX4 (Fig. 1*B*). Sequential labeling of cysteines with *N*-ethyl maleimide (NEM) at the time of lysis, followed by reduction with DTT, and then iodoacetamide labeling of newly formed thiols allowed for the identification of changes in protein oxidation by MS/MS (Fig. 1*C*). MS/MS showed that in control cells that were not treated with TBuOOH, the peptide containing the catalytic cysteine (Cys127) was only identified with NEM bound, indicating that at the time cells were harvested it was reduced (Fig. 1, *D*–*G*). However, the peptide containing Cys127 in the HMW PRDX4 species from cells exposed to TBuOOH showed oxidation to a sulfonic acid (−SO_3_) at the Cys127 residue (Fig. 1, *D* and *G*). These results demonstrate that the HMW species of PRDX4 found in oxidizing conditions contain primarily an overoxidized peroxidatic cysteine and that the catalytic cysteine in the monomeric form of PRDX4 is only found in the reduced state.

### Oxidation of PRDX4 alters its binding partners

**Figure 2:**
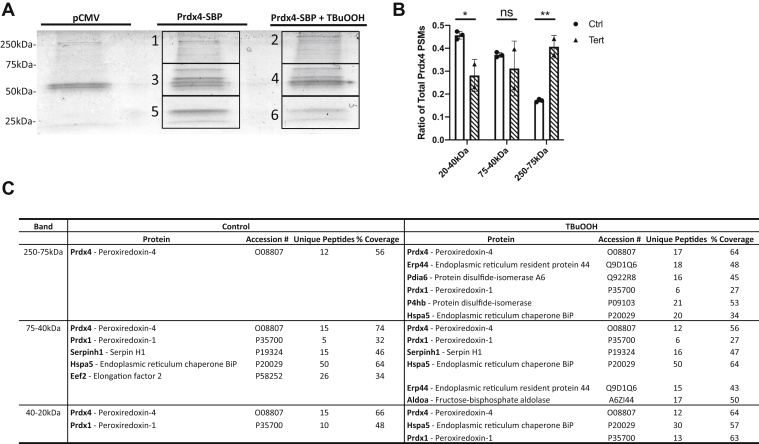
**Oxidation of PRDX4 alters its binding partners.***A*, Coomassie blue gel of the pulldown of PRDX4-SBP from C10 cell lysates treated with vehicle control or 500 μM TBuOOH for 2 min. Bands cut out for further analysis by MS are indicated. *B*, peptide spectral matches for PRDX4 identified in each band compared with total PRDX4 peptide spectral matches identified in each sample. *C*, MS/MS results showing the proteins found in each band. MS/MS, tandem MS; PRDX4, peroxiredoxin-4; TBuOOH, tertbutyl hydroperoxide.

We next sought to determine if the oxidation of PRDX4 alters its' interacting proteins and if there is a dependence on the oligomeric structure of PRDX4 for these interactions. Expression of PRDX4-SBP in C10 cells allowed for the detection of PRDX4-binding partners in monomer, dimer, and HMW forms *via* MS/MS. PRDX4 complexes were coimmunoprecipitated from C10 cells expressing PRDX4-SBP, treated with or without TBuOOH, and subjected to nonreducing SDS-PAGE (Fig. 2*A*). Spectral counts of PRDX4 found in each band roughly reflected band intensities in Western blots and showed increases in PRDX4 contained in HMW species upon oxidation (Fig. 2*B*). To ensure the validity of the identified proteins, we used a rigorous filtering methodology whereby only proteins not identified in control transfected cells and identified in all three replicate experiments with SEQUEST HT scores (Thermo Fisher Scientific) above 100 in at least two of the replicates are shown (Fig. 2*C* and Fig. S1). Of the nine detected proteins pulled down with PRDX4, six of them are ER-localized proteins. Oxidation of PRDX4 with TBuOOH showed an increase in proteins associating with the HMW species compared with dimer or monomer (Fig. 2*C*). In the vehicle control–treated cells expressing PRDX4-SBP, no other proteins were found to be associated with PRDX4 in the 250 kDa region. In the TBuOOH-treated group, six proteins, PRDX1, HSPA5, TXNDC5, ERP44, P4HB, and PDIA6, were pulled down with HMW PRDX4 (Fig. 2*C*) in three independent experiments. In the region of 40 to 75 kDa, in control cells, PRDX1 and eukaryotic elongation factor 2 were found to interact with PRDX4, whereas in this molecular weight (MW) range, in TBuOOH-treated cells, eukaryotic elongation factor 2 was no longer detected while in addition to PRDX1, TXNDC5, ERP44, and ALDOA were also pulled down with PRDX4. Finally in the MW range of 20 to 40 kDa, PRDX1 was found to interact with PRDX4 in control cells, while in response to TBuOOH, in addition to PRDX1, HSPA5 also was associated with PRDX4 (Fig. 2*C*). Previous studies had already identified ERP44, PDIA6, and P4HB as binding partners for PRDX4 (20, 21). However, in this study, we found that interactions of PRDX4 with various proteins depended on PRDX4 oxidation and oligomeric state. Finally, the demonstration that multiple proteins interact with HMW species of PRDX4 provide a possible explanation for the multiple bands seen in the HMW species of PRDX4 in Figure 1*A*.

### Recombinant PRDX4 forms HMW oligomers when oxidized independently of interacting proteins

**Figure 3:**
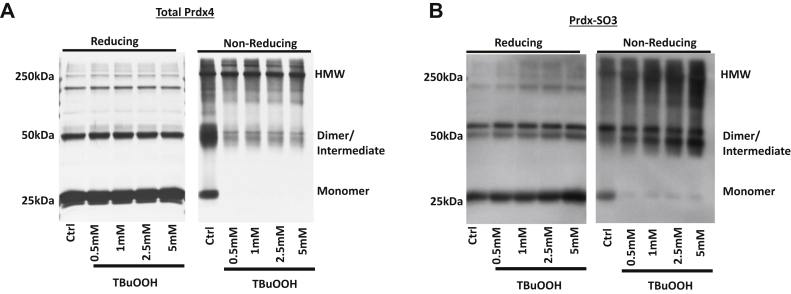
**Recombinant PRDX4 forms HMW species when overoxidized.** Recombinant human PRDX4 was reduced with 10 mM DTT for 30 min. Subsequently, the DTT was removed *via* spin column, and the rhPRDX4 was treated with 10 mM DTT or 1 mM TBuOOH for 30 min. Western blots were run reducing and nonreducing. *A*, Coomassie-stained blot showing total rhPRDX4. *B*, Western blot with overoxidized PRDX antibody showing overoxidized PRDX4 (PRDX4-SO_3_). HMW, high molecular weight; PRDX4, peroxiredoxin-4; TBuOOH, tertbutyl hydroperoxide.

To determine if the formation of PRDX4 HMW species observed under nonreducing conditions was dependent on the binding of interacting proteins, recombinant human PRDX4 was oxidized using TBuOOH. Recombinant PRDX4 ran primarily as a monomer on a reducing SDS-PAGE gel, along with a dimer band, which could not be reduced with DTT under the conditions used herein (Fig. 3*A*). Nonreducing SDS-PAGE showed that TBuOOH caused a shift toward HMW species and a concomitant loss of monomer and dimer PRDX4 species. Western blotting for PRDX-SO_3_ confirmed the oxidation of recombinant PRDX4 with the addition of TBuOOH, and under nonreducing conditions, this overoxidation corresponded to the dimer and HMW band (Fig. 3*B*). These data further confirm that when PRDX4 is overoxidized to a sulfonic acid, it promotes formation of dimeric and HMW species, and this shift can happen independently of PRDX4-interacting proteins.

### Catalytic cysteines in PRDX4 play important role in HMW formation

**Figure 4:**
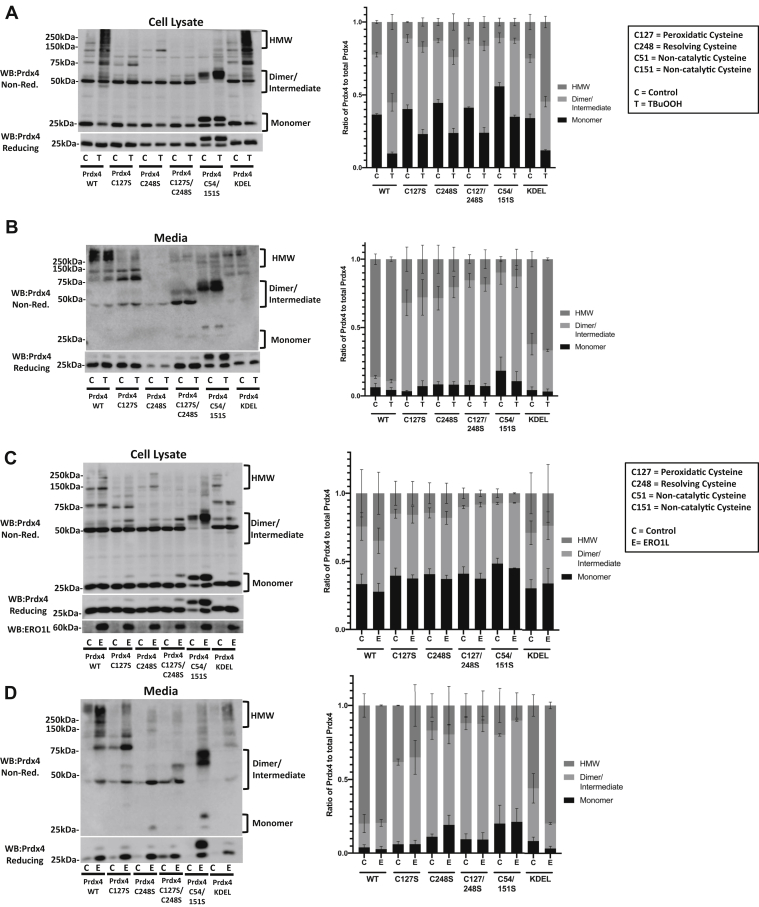
**Cysteines in PRDX4 play an important role in PRDX4 HMW species formation.***A*, reducing and nonreducing Western blots for PRDX4 in C10 cells expressing PRDX4 mutant constructs treated with vehicle control (C) and 500 μM TBuOOH (T) for 2 min. *B*, reducing and nonreducing Western blots for PRDX4 in the supernatant from C10 cells expressing PRDX4 mutants. *C*, reducing and nonreducing Western blots for PRDX4 and ERO1a in C10 cell lysates expressing PRDX4 mutant constructs and either pCMV (C) or ERO1a (E). *D*, reducing and nonreducing Western blots for PRDX4 in the supernatant from C10 cells expressing PRDX4 mutants and either pCMV (C) or ERO1a (E) for 24 h. *Right panels*, quantification of the three distinct PRDX4 species in the bracketed regions in the nonreducing Western blots compared with total PRDX4 detected in these fractions combined. Results are mean plus SEM values derived from multiple pooled experiments. ERO1a, endoplasmic reticulum oxidoreductase alpha; HMW, high molecular weight; PRDX4, peroxiredoxin-4; TBuOOH, tertbutyl hydroperoxide.

The role of cysteines in PRDX4 oligomerization has not been previously studied, and the involvement of the active-site cysteines in PRDX4 to regulate oxidation-dependent shifts in oligomerization remains unclear. We next explored which cysteines of PRDX4 contributed to the formation of HMW species. Mutation of the two cysteines involved in the catalytic cycle caused an alteration in the HMW PRDX4 complexes when oxidized with TBuOOH (Fig. 4*A*). Notably, mutation of the peroxidatic cysteine (Cys127) or the resolving cysteine (Cys248) individually caused a loss of HMW species in cells exposed to TBuOOH and resulted in the appearance of an apparent intermediate MW species under nonreducing conditions. The mutation of both Cys127 and Cys248 largely abrogated any differences in the banding pattern in cells exposed to TBuOOH (Fig. 4*A*). Simultaneous mutation of the noncatalytic cysteines (Cys54 and Cys151) led to the appearance of a second monomeric PRDX4 band at approximately 30 kDa present under nonreducing or reducing conditions, which possibly indicate incomplete cleavage of the signal peptide of PRDX4. The mutation of Cys54 and Cys151 also led to the formation of PRDX4 in the intermediate range, which was increased upon treatment with TBuOOH. Simultaneous mutation of Cys54 and Cys151 attenuated the TBuOOH-induced formation of HMW complexes (Fig. 4*A*).

As previously mentioned, PRDX4 is also a secreted protein in certain conditions (22). Analysis of the media from cells overexpressing the PRDX4 constructs revealed that WT PRDX4 found in the media was almost exclusively present as an HMW species under nonreducing conditions. No differences were apparent in the amount or MW of WT PRDX4 found in the media in the TBuOOH-treated cells compared with the vehicle control (Fig. 4*B*). Mutation of Cys127 showed similar levels in the media as WT PRDX4 but mostly as intermediate MW (<150 kDa) species (Fig. 4*B*). In contrast, less Cys248Ser mutant PRDX4 was found in the media, compared with WT or Cys127Ser PRDX4, suggesting that the resolving cysteine may play a role in regulating PRDX4 intracellular retention and/or secretion. However, the Cys248 mutant was expressed at slightly lower levels, which could have affected the amount of secreted C248S PRDX4. Finally, mutation of both Cys127 and Cys248 together did not affect secretion of PRDX4 compared with WT PRDX4, although the MW of this secreted mutant PRDX4 was consistent with that of a dimer, instead of the HMW size of secreted WT PRDX4. PRDX4 lacks an ER retention sequence and is retained in the ER through disulfide exchange with ERP44 as well as PDI (23). We next determined whether the addition of a KDEL sequence added to the C terminus of PRDX4 affected cellular retention, secretion, or HMW formation. As anticipated, KDEL-PRDX4 showed TBuOOH-induced HMW formation similar to WT PRDX4 (Fig. 4*A*). However, KDEL-PRDX4 showed markedly diminished secretion into the supernatant, compared with WT PRDX4 (Fig. 4*B*). These findings demonstrate that the absence of an ER retention sequence in PRDX4 contributes to its secretion under conditions of oxidative stress.

TBuOOH induces oxidation in a nontargeted manner. We therefore sought to investigate the impact of ER-targeted oxidant formation for HMW species formation and secretion of PRDX4. We therefore overexpressed endoplasmic reticulum oxidoreductase alpha (ERO1a) to specifically induce H_2_O_2_ formation within the ER. While the formation of HMW species in WT PRDX4 in cell lysates was modest compared with the TBuOOH treatment (Fig. 4*C* compared with Fig. 4*A*), more HMW PRDX4 was found in supernatants in cells expressing ERO1a (Fig. 4*D*). Expression of ERO1a also increased the overall amount of PRDX4 in the media, and this was observed following coexpression of WT or cysteine mutants of PRDX4 (Fig. 4*D*). Coexpression of ERO1a with PRDX4-C127S and PRDX4-C248S mutants showed formation of intermediate MW complexes in cells and supernatants (Fig. 4, *C* and *D*), consistent with patterns observed in response to TBuOOH (Fig. 4, *A* and *B*). The mutation of both the noncatalytic cysteines in PRDX4 (Cys54 and 151) resulted in the formation of PRDX4 species of intermediate MW, both under nonreducing and reducing conditions indicative of improper assembly or folding of this mutant PRDX4 molecule. Following exposure to TBuOOH or overexpression of ERO1a, virtually no HMW species of PRDX4 Cys54/151 mutant were observed (Fig. 4, *A*–*D*), indicating an involvement of noncatalytic cysteines in folding of PRDX4 and HMW complex formation. Finally, adding a KDEL sequence to PRDX4 greatly reduced the amount of PRDX4 found in the media of cells coexpressing ERO1a (Fig. 4*D*), similar to observations with TBuOOH (Fig. 4*B*). Collectively, these results show the contribution of all four cysteines of PRDX4 to the formation of HMW complexes, the importance of catalytic cysteines, and the absence of an ER retention sequence for the secretion of PRDX4 under oxidizing conditions.

### Mutation of residues important in PRDX overoxidation and decamer stability alter the ability of PRDX4s to form HMW species

**Figure 5:**
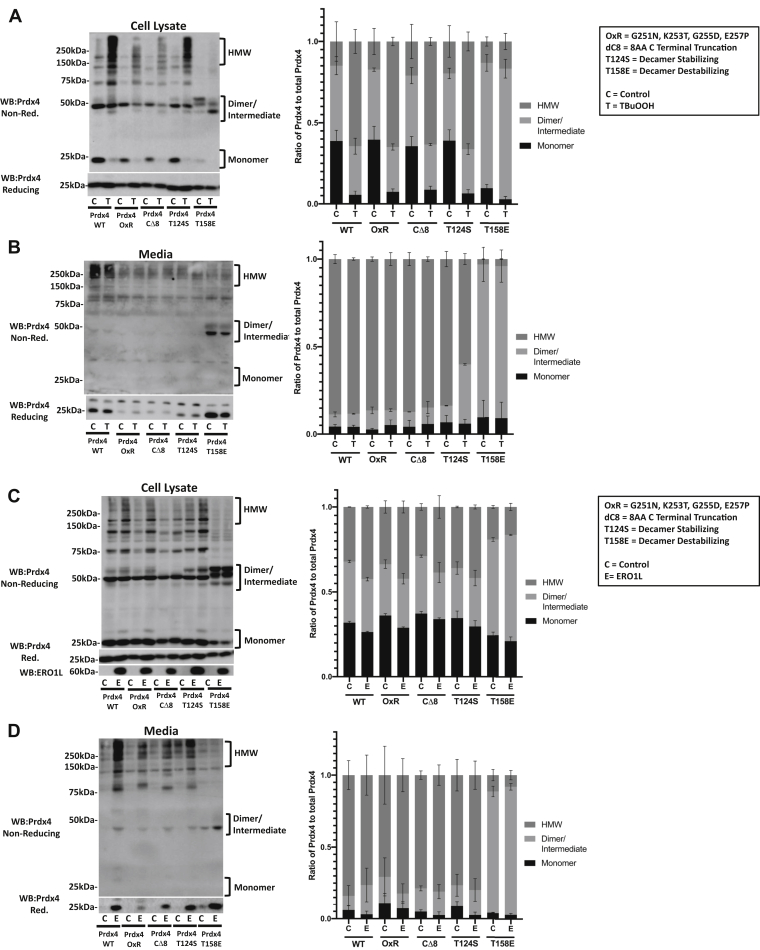
**Oxidation-resistant, decamer-promoting, and decamer-resistant mutants alter PRDX4 HMW species.***A*, reducing and nonreducing Western blots for PRDX4 in C10 cells expressing PRDX4 mutant constructs treated with vehicle control (C) and 500 μM TBuOOH (T) for 2 min. *B*, reducing and nonreducing Western blots for PRDX4 in the media from C10 cells expressing PRDX4 mutants. *C*, reducing and nonreducing Western blots for PRDX4 and ERO1a in C10 cell lysates expressing PRDX4 mutant constructs and either pCMV (C) or ERO1a (E). *D*, reducing and nonreducing Western blots for PRDX4 in the media from C10 cells expressing PRDX4 mutants and either pCMV (C) or ERO1a (E) for 24 h. *Right panels*, quantification of the three distinct PRDX4 species in the bracketed regions in the nonreducing Western blots, compared with total PRDX4 detected in these fractions combined. Results are mean plus SEM values derived from multiple pooled experiments. ERO1a, endoplasmic reticulum oxidoreductase alpha; HMW, high molecular weight; PRDX4, peroxiredoxin-4; TBuOOH, tertbutyl hydroperoxide.

Each of 2-cys PRDX family members found in eukaryotes have a different susceptibility to overoxidation (24), and bacterial PRDX analogs have been shown to be far less susceptible to overoxidation than mammalian PRDXs (25). A wide range of models have been published to explain how the sensitivity of different PRDXs is affected based on their tertiary structure and amino acid sequences (24, 25, 26, 27, 28). We next sought to determine the effect of similar mutations in PRDX4 to assess their impact on the susceptibility of PRDX4 to oligomerization and secretion. Human PRDX3 has been shown to be less susceptible to overoxidation compared with the other 2-cys PRDXs (26, 27). Studies have shown that mutation of four amino acids downstream of the resolving cysteine of PRDX2 to match the PRDX3 sequence causes PRDX2 to become less susceptible to overoxidation (28). Based on the latter findings, we mutated the same four amino acids downstream of the resolving cysteine in mouse PRDX4 (G251N, K253T, G255D, and E257P) to match the sequence of PRDX3 and created a PRDX4 construct that was potentially more overoxidation resistant (referred to hereafter as PRDX4-OxR). In cells expressing PRDX4-OxR, we did not observe any marked differences in the pattern of oligomerization of PRDX4 after treatment with TBuOOH or in cells coexpressing ERO1a, when compared with WT-PRDX4, although the intensity of HMW species was decreased in response to TBuOOH but not ERO1a (Fig. 5, *A* and *C*). However, G251N, K253T, G255D, and E257P mutant PRDX4 decreased secretion of the PRDX4 mutant into the media, as there was a marked decrease in the amount of PRDX4-OxR found in the supernatant compared with WT-PRDX4 (Fig. 5, *B* and *D*).

Bacterial PRDX analogs, which are highly resistant to overoxidation, are lacking a C-terminal alpha helix that contains a conserved tyrosine and phenylalanine (“YF”) motif that is conserved in mammalian 2-cys PRDXs (25). Structural studies have shown that this “YF” loop must undergo local unfolding and move out of the way before the disulfide bond can form between the oxidized peroxidatic cysteine and the resolving cysteine (25). The loss of the “YF” motif allows the disulfide bond to form faster and reduces the likelihood that another H_2_O_2_ molecule will be able to further oxidize the peroxidatic cysteine to a sulfinic acid before the disulfide bond is formed. Mutation studies have shown that the C-terminal truncation to remove the a7 alpha helix and the “YF” motif promotes resistance to overoxidation. We therefore truncated PRDX4 by eight amino acids on the C terminus to remove the “YF” motif (PRDX4-CΔ8) and addressed HMW formation and secretion upon oxidation. In cells exposed to TBuOOH, or expressing ERO1a, overall PRDX4-CΔ8 HMW species tended to be decreased both in cells and supernatants (Fig. 5, *A*–*D*), although no clear differences in the ratio of HMW species formed upon oxidation were detected as compared with WT PRDX4 (Fig. 5, *A*–*D*).

The mutation of the threonine residue in close proximity to the peroxidatic cysteine to serine in *Salmonella typhimurium* alkyl hydroperoxide reductase subunit C (Thr43) and *Saccharomyces cerevisiae* thiol-specific antioxidant 1 (Thr44) stabilizes the decamer formation, while not affecting the activity (29, 30). To assess how alteration of the active site affects mouse PRDX4 oligomerization, we therefore mutated the corresponding threonine (Thr124) to serine. A nonreducing Western blot showed that PRDX4-T124S displayed no clear differences neither in HMW species at steady state or after TBuOOH treatment (Fig. 5*A*) nor in cells coexpressing ERO1a (Fig. 5*C*). These findings indicate that PRDX4-T124S does not display the same level of oligomer stabilization as has previously been shown with *S. cerevisiae* thiol-specific antioxidant (27) and more closely resembles results observed with *S. typhimurium* alkyl hydroperoxide reductase subunit C (29).

Aromatic residues on the decamer-binding interface have been shown to be important in PRDX decamer formation (31). In order to assess how adding a charged residue to the decamer-binding interface would affect the oligomerization of PRDX4 in response to TBuOOH, we mutated threonine 158, which is located on the decamer-binding interface of PRDX4. The mutation of threonine to a glutamic acid residue causes the PRDX4 dimers to be repelled from each other by the negative charge on both dimer-binding interfaces and does not allow for decamers to form (32). Results in Figure 5, *A* and *C* demonstrate a decrease of PRDX4 monomer as well as HMW species in control cells expressing the T158E mutation, along with an appearance of three distinct bands that form around 50 kDa. In response to TBuOOH, T158E PRDX4 migrated faster under nonreducing conditions indicative of an overoxidized dimer with an increased negative charge, which could explain the increased migration of the T158E PRDX4 dimer on the gel. No clear differences in the migration of T158E PRDX4 were observed in cells coexpressing ERO1a (Fig. 5*C*). However, expression of T158E PRDX4 resulted in more secretion into the supernatant, as compared with WT PRDX4 (Fig. 5, *B* and *D*). In aggregate, these findings show that various domains that govern susceptibility of overoxidation and decamer formation of other PRDX proteins have a varying impact in the formation of HMW species and/or secretion of PRDX4.

## Discussion

PRDX4 is emerging as an important therapeutic target in numerous diseases, such as metabolic syndromes (33), idiopathic pulmonary fibrosis (IPF) (34), liver injury (35), amyloid-beta aggregate-induced neuronal apoptosis (36). PRDX4 is a complex protein as it can form multiple oligomeric structures based on its oxidation status, can shift from having peroxidase function to acting as a chaperone, and is localized either in the ER or extracellularly. Therefore, a better understanding of the mechanisms that control PRDX4 oxidation, function, and subcellular localization along with the elucidation of proteins binding to PRDX4 will be important for uncovering the role of PRDX4 in the aforementioned diseases.

Recent advances have allowed for real-time monitoring of PRDX oligomerization and have shown that there are differences in oligomeric structure dynamics of typical 2-cys PRDXs when overoxidized (37); however, the dynamics of PRDX4 oligomerization in response to oxidation had not previously been investigated. In the present study, we confirmed that the overoxidation of PRDX4 leads to HMW oligomeric structures of PRDX4 using MS and demonstrated that the peroxidatic cysteine of these HMW PRDX4 species was overoxidized to the sulfonic acid (−SO_3_) form. Our findings of PRDX4 HMW complex formation with overoxidation fit with previous reports for other 2-cys PRDX family members (8, 38, 39). Confirmation of the sulfonic modification of the peroxidatic cysteine in HMW species of PRDX4 elucidates that with the oxidation, PRDX4 transitions from monomers and dimer to HMW species, concomitant with an oxidation-dependent shift from peroxidase activity to chaperone activity. The chaperone activity of overoxidized PRDX remains ill defined, and in this study, we demonstrated differences in the binding partners of PRDX4 under nonreducing steady state conditions and when PRDX4 is present as overoxidized HMW species, findings that will help to elucidate how the shift from peroxidase to chaperone alter PRDX4s cellular function. Furthermore, the susceptibility of PRDX4 to oxidation by different peroxides is not currently known. In the present study, TBuOOH was used as it has been previously shown to be a potent inactivator of the peroxidase activity of PRDX4s (19). However, further studies are needed to determine the role of other peroxides besides TBuOOH in PRDX4 inactivation, oligomerization, and binding interactions.

PRDX4 has been implicated in a redox relay where it undergoes a disulfide exchange with PDI in the ER, forming a H_2_O_2_-dependent PDI recycling pathway (20, 21). However, a broad screen of PRDX4-interacting partners had not been completed, and the role that the quaternary structure and oxidation status of PRDX4 play in protein interactions had yet to be studied. In this article, we show that the structure of PRDX4 governs its binding partners, as we identified different client proteins interacting with the monomeric, dimeric, or HMW PRDX4 species under nonreducing conditions. A number of the interacting proteins identified in this study, notably ERP44, TXNDC5, and the PDIs, P4HB and PDIA6, have been previously shown to interact with PRDX4 (20). PDI family members appear to interact preferentially with the oxidized forms of PRDX4, with P4HB and PDIA6 only being detected in the HMW region (75–250 kDa) of the TBuOOH-treated cells. These findings fit with the previously shown disulfide exchange between some PDI family members and PRDX4 (20), as increased oxidation of PRDX4 would increase the amount of PRDX4 species with disulfide bonds between the peroxidatic and resolving cysteines and allow for increased disulfide exchange with the family of PDIs. The interaction of PDIs with only the HMW species could indicate a complex of PDIs interacting with PRDX4 decamers at once or preferential binding to PRDX4 when it is in the oligomeric state. Further studies will be needed to determine the exact stoichiometry of PRDX4 subunits involved in the formation of HMW species complexes and the nature of the molecular interaction(s) between PRDX4 and the interacting proteins identified herein. Another notable protein interacting with PRDX4 in response to TBuOOH in the low MW and HMW regions was HSPA5, a master regulator of the unfolded protein response (UPR) in the ER. There is no prior link between PRDX4 and HSPA5, and the mechanisms whereby ER “redox” stress may regulate the UPR are not currently well understood. It is possible that an increase in oxidants within the ER lead to oxidation of PRDX4, thus promoting the binding of PDIs and HSPA5, which could trigger the ER stress response. Further targeted studies investigating ER oxidation will be needed to determine whether an ER-specific oxidation imbalance and/or PRDX4 overoxidation act as inducers of the UPR and downstream signaling cascades.

Of note, PRDX1 also was found as a PRDX4-interacting protein that was detected both in control and TBuOOH-exposed cells and that associated with PRDX4 monomer, dimer, and HMW species. The interaction between PRDX1 and PRDX4 has been previously shown; however, the question around the localization of this interaction of cytosolic PRDX1 and the ER-localized PRDX4 remains to be resolved. The lack of a KDEL ER retention sequence could lead to a portion of PRDX4 to be present in the cytosol. In the present study, wherein we overexpressed PRDX4, it is possible that the ERP44 retention mechanism was overwhelmed leading to some PRDX4 to leak into the cytosol where it could come in contact with PRDX1. Interestingly, both PRDX1 and PRDX4 have been shown in separate studies to be released form cells in extracellular vesicles (22, 40), so it remains possible that the two PRDXs interact as they are being packaged into extracellular vesicles for release from the cells.

Herein, we generated a number of PRDX4 mutants in order to gain insights into the interplay between oxidation of specific PRDX4 cysteines and PRDX4 oligomerization state upon oxidation with TBuOOH. Oxidation of PRDX4 cysteine mutants gives insight into which PRDX4 MW species present under nonreducing conditions are dependent on the peroxidatic (Cys127), resolving (Cys248), or the noncatalytic cysteines (Cys54 and Cys151). Mutation of the peroxidatic cysteine (Cys127) to serine caused a loss of HMW species as well as the loss of a band at an apparent MW of ~150 kDa. This implies that the peroxidatic cysteine is important for the formation of the 150 kDa band that is present in the control and oxidized WT-PRDX4 lanes. Alternatively, the resolving cysteine (Cys248) to serine mutation did not affect the 150 kDa band but caused the loss of the ~75 kDa band. It is unclear if the difference in the bands seen reflect oligomers of PRDX4 or are caused by the loss of interaction with client proteins. The changes in MW observed under nonreducing conditions in cells expressing the Cys54/151 mutant are possibly because of a defect in protein processing, as the additional 30 kDa band seen in the reducing Western blot matches the MW of the full-length PRDX4 protein where the signal peptide is not cleaved off, leading to a 30 kDa band as well as the typical 27 kDa PRDX4 band (41).

The mechanisms leading to the secretion of PRDX4 remain largely unknown. A study by Lipinski *et al.* (22) showed that upon LPS treatment, PRDX4 was secreted from cells *via* extracellular vesicles. Western blots under nonreducing conditions of isolated extracellular vesicles revealed PRDX4 dimers and HMW species, similar to observations in the present study. PRDX4 has been reported in the serum of patients with diabetes (42) and IPF (34). Interestingly, both these diseases are linked with ER stress (43, 44), pointing to a possible link between ER stress and PRDX4 secretion. The discovery of HSPA5, an initiator of the ER stress response, as a binding partner of oxidized PRDX4 oligomers adds more credence to a possible link between PRDX4 oxidation and ER stress responses.

Our studies examining PRDX4 overoxidation-resistant mutants (OxR and CΔ8) and their effect on PRDX4 oligomerization showed small decreases in intracellular PRDX4 HMW species and decreases in HMW PRDX4 species in supernatants. The proposed faster reaction mechanisms of the oxidation-resistant mutants could enhance their ability to interact with other client proteins to promote intracellular retention. The increased rate of resolution form sulfenic acid to disulfide bond formation may allow for more time for proteins such as ERP44 or PDI to interact with PRDX4 and thus promote the retention within the ER. In the case of ERO1a overexpression, it is possible that enhanced interaction between PRDX4 and client proteins other than ERP44 led to the increased cellular retention of PRDX4. As expected, the addition of a negatively charged residue to the decamer-binding face led to ablation of PRDX4 HMW species, even under oxidizing conditions. The banding pattern around 50 kDa is indicative of an overoxidized dimer species. The demonstration that inhibition of decamer formation leads to increase in the amount of PRDX4 found in the media in cells exposed to TBuOOH or expressing ERO1a points to a role of PRDX4 decamer formation in promoting retention of PRDX4, perhaps because of the interaction with ER chaperone proteins that associate with HMW PRDX4 complexes. Finally, one limitation of the present study is that it is based on overexpression of PRDX4 constructs. It is not clear whether the presence of endogenous PRDX4 interfered with the function of mutant PRDX4 constructs. Furthermore, the overexpression of PRDX4 might also have overwhelmed ER chaperones. Further studies that employ conditional ablation and mutagenesis strategies will be required to alleviate this concern.

In the present study, we provide new insights into the factors that govern PRDX4 HMW formation and secretion in settings of oxidative stress. Additional studies also will be needed to monitor the subcellular trafficking of WT and mutants of PRDX4, mechanisms that govern its ER retention *versus* secretion. Furthermore, the potential (patho)physiological role of extracellular overoxidized PRDX4 in regulating stress responses, potentially as a “danger” signal, also warrants further investigation. The demonstration that the chaperone, HSPA5, a critical regulator of ER stress responses, binds PRDX4 under oxidizing conditions points to a putative role of PRDX4 oxidation in triggering ER stress responses, a possibility that will require further investigation. Such studies have the potential to elucidate the importance of disruptions in the ER redox environment for the pathogenesis of diseases such as diabetes and IPF where between increased ER stress, oxidative stress and PRDX4 secretion have already been demonstrated.

## Experimental procedures

### Chemicals and reagents

All chemicals utilized were purchased from Millipore-Sigma unless otherwise noted. Antibodies: anti-PRDX4 (ab59542) from Abcam and anti-PRDX-SO_3_ (LF-PA0004) from AbFrontier. Concerns exist regarding specificity of PRDX antibodies. The PRDX4 antibody used herein was validated by siRNA-mediated knockdown of PRDX4 in C10 cells (data not shown) as well as in a previously published *Prdx4* knockout mouse model (22).

### Cell culture

Mouse lung epithelial cells (C10) were cultured in Connaught Medical Research Laboratories media (Gibco; 11530-037) supplemented with 10% fetal bovine serum (Gibco; 16000-044) with 2 mM l-glutamine (Gibco; 25030-081). C10 cells were validated through confirmation of expression of *Sftpc* and *Abca3* mRNA and were previously authenticated (45). C10 cells were transfected with one of the *PRDX4* plasmids or an empty vector (pCMV) by incubation with the 500 ng plasmid and Lipofectamine 2000 (Invitrogen) for 4 h in serum-free media. Subsequently, the C10 cells were placed back into full media with fetal bovine serum and allowed to recover overnight.

### Creation of PRDX4 mutants

Untagged WT mouse Prdx4 plasmid was obtained from Origene (MC201312). Primers for creating Prdx4 mutants were designed using the QuickChange Primer Design tool (Agilent) and ordered from Integrated DNA Technologies. PRDX4 mutants were created using the QuickChange Site-Directed Mutagenesis Kit (Agilent; 200519). Plasmids were purified using the EndoFree Plasmid Maxi Kit (Qiagen; 12362). All plasmids were validated by Sanger sequencing.

### Recombinant human PRDX4 oxidation

Recombinant human PRDX4 (5 ng protein; ab93947; Abcam) was reduced using 10 mM DTT for 30 min at room temperature. The protein was then passed through a BioSpin column (Bio-Rad) to remove the DTT from the reduced protein. The reduced protein was then treated with 1 mM TBuOOH for 30 min at room temperature and loaded onto an SDS-PAGE gel, run with or without DTT.

### Identification of overoxidized PRDX4 and PRDX4-binding partners *via* MS/MS

C10 cells grown in 10 cm dishes were transfected with pCMV or PRDX4-SBP constructs. About 24 h after transfection, cells were treated with 500 μM H_2_O_2_ or TBuOOH for 2 min before being harvested in coimmunoprecipitation buffer (20 mM Tris, pH 7.4, 150 mM NaCl, 0.5% tergitol-type NP-40, 10% glycerol, 20 mM NEM, and 200 U catalase). PRDX4 was then immunoprecipitated from 1.5 mg total protein with high-affinity streptavidin agarose beads (Invitrogen) as described previously (46). PRDX4 and bound proteins were eluted using 4 mM biotin in Tris-buffered saline and run on a nonreducing SDS-PAGE gel.

The gel was stained with Coomassie blue, the protein was excised from the gel, and the gel bands were washed with 50% methanol and 5% acetic acid to remove the Coomassie blue stain. The protein was reduced with 10 mM DTT and alkylated with 100 mM iodoacetamide followed by digestion with Promega sequencing grade modified trypsin (20 ng/μl), (V5113; Promega), for 16 h (Promega). The digested samples were analyzed on the Q-Exactive Plus mass spectrometer coupled to an EASY-nLC 1200 (Thermo Fisher Scientific). MS data were acquired in a data-dependent acquisition mode with lock mass function activated in which a survey scan from *m*/*z* 350 to 1600 at 70,000 resolution was followed by 10 higher-energy collisional dissociation MS/MS scans on the most abundant ions at 17,500 resolution. The data-dependent data were analyzed using Proteome Discover 2.4 and SEQUEST software for protein identification within the uniprot_ref_mouse database containing 63,368 sequences (downloaded on November 2019). Spectra with more than two missed cleavages were removed from the analysis. Mass tolerance of precursor ions was 10 ppm, and mass tolerance for fragment ions was 0.02 Da. Target Decoy PSM Validator was included in the workflow to limit the false discovery rate to less than 1%. Variable modification of methionine oxidation, cysteine alkylation with iodoacetamide and NEM, and cysteine oxidation were considered. All protein identification results were filtered to include only high confidence peptides and a minimum of two unique peptides per protein. Identified proteins were filtered for detection in all three repeated experiments with a SEQUEST HT score greater than 100 in at least two experiments.

## Data availability

All experimental data for this article are available upon e-mail request to Yvonne Janssen-Heininger (University of Vermont; yvonne.janssen@med.uvm.edu). The raw data for the MS analysis following the pulldown of PRDX4-SBP can be found in Fig. S1 and has been uploaded to the PRIDE repository (project accession identifier: PXD024687).

## Supporting information

This article contains supporting information.

## Conflict of interest

The authors declare that they have no conflicts of interest with the contents of this article.
